# Aberrant Myelopoiesis and Haematopoietic Stem Cell Niche Remodelling Precedes Cancer Cachexia Onset in a Sex‐Dependent Manner

**DOI:** 10.1002/jcsm.70403

**Published:** 2026-10-16

**Authors:** Alice Tate, Alex Brown, Lisa Ek Orloff, James Vanhie, Nicolas Collao, Cooper Brabrook, Victoria Moghadam, Vedrana Rajaković, Nadine Wiper‐Bergeron, Michael De Lisio

**Affiliations:** ^1^ School of Human Kinetics, Faculty of Health Science University of Ottawa Ottawa Canada; ^2^ Department of Cellular and Molecular Medicine, Regenerative Medicine Program University of Ottawa Ottawa Canada; ^3^ Éric Poulin Centre for Neuromuscular Disease University of Ottawa Ottawa Canada

**Keywords:** bone marrow, CCR2‐CCL2 signalling, haematopoietic stem cells, inflammation, Lewis lung carcinoma, muscle wasting

## Abstract

**Background:**

Male cancer patients are 30% more likely to develop cachexia compared to females; however, the underlying aetiology remains unknown. Myelopoiesis induces inflammation in cancer cachexia, which contributes to muscle atrophy. Whether differences in myelopoiesis during cancer cachexia initiation contribute to sex‐based differences in cachexia incidence remains unknown.

**Methods:**

Male and female C57BL/6 and B6(C)‐Ccr2^tm1.1Cln^/J mice were randomized into sham (PBS) or were inoculated subcutaneously with Lewis lung carcinoma (LLC) to induce cachexia. Mice were sacrificed 2 (14 days), 2.5 (17 days) and 3.5 weeks (24 days) post‐inoculation. Changes in body composition, haematopoietic stem and progenitor cells (HSPCs), myeloid immune cell migration, bone marrow niche cellular dynamics and CCL2 expression were assessed.

**Results:**

LLC inoculation reduced tumour‐free body weight, lean mass, gastrocnemius–soleus mass and myofiber cross‐sectional area (CSA) in males at 3.5 weeks (*p* < 0.05), while females maintained tumour‐free body weight, lean mass and CSA. Within the bone marrow, haematopoietic stem cells (HSCs) were increased at 2, 2.5 and 3.5 weeks compared to sham in males and at 2.5 weeks compared to 2 and 3.5 weeks in females (*p* < 0.05). Multipotent progenitors (MPPs) were significantly increased at 2, 2.5 and 3.5 weeks compared to sham in males and at 2.5 weeks compared to sham, 2 and 3.5 weeks in females (*p* < 0.05). Downstream, common myeloid progenitors (CMPs) and granulocyte‐monocyte progenitors (GMPs) were elevated in males at 2.5 weeks, with CCR2^+^GMPs peaking at 2.5 weeks in males and 3.5 weeks in females (*p* < 0.05). Sex differences in HSC niche composition were also observed with osteoprogenitors, osteoblasts and endothelial cells increased at 2 weeks in males, while females showed increased osteoprogenitors and endothelial progenitors at 2.5 weeks in tumour‐bearing mice (*p* < 0.05). Plasma C‐C motif chemokine ligand 2 (CCL2) concentrations increased with tumour progression in both sexes; however, CCR2^+^‐progenitors and macrophages were decreased at 2.5 and 3.5 weeks compared to 2 weeks (*p* < 0.05). In skeletal muscle, CCL2 concentration increased at 3.5 weeks, and the proportion of CCR2^+^/CD206^−^ macrophages increased, while total macrophage abundance remained unchanged (*p* < 0.05). Macrophage subpopulations were related to decreasing CSA in males but not females.

**Conclusion:**

Our study suggests that aberrant myelopoiesis and sex‐specific HSC niche remodelling precede muscle atrophy during cachexia development in males. These findings provide an important first step in understanding the role aberrant myelopoiesis plays in cancer cachexia and establish myeloid skewing as a potential therapeutic target.

## 1 Introduction

Cancer cachexia, the continuous skeletal muscle and fat loss experienced by 50%–80% of cancer patients, leads to diminished functional capacity, decreased tolerance to cancer treatments, and directly contributes to over 30% of cancer‐associated mortalities [1]. Biological sex influences cachexia prevalence as males are twice as likely to develop cachexia compared to females [2]. Males exhibit reduced muscle strength, with a correlation between muscle mass and cancer‐related fatigue that is absent in females [3]. Cachexia is a chronic inflammatory condition characterized by higher circulating levels of pro‐inflammatory cytokines, including IL‐1, IL‐6, TNF‐α and INFγ [4]. Chronic inflammation promotes tumorigenesis [5] while simultaneously contributing to skeletal muscle atrophy by suppressing protein synthesis pathways and enhancing proteolysis [6]. While both males and females have elevated pro‐inflammatory cytokine levels in cachexia, their function differs by sex. In females, IL‐6 overexpression does not induce or accelerate cachexia but correlates with elevated free fatty acid levels, whereas in males, an IL‐6 antagonist prevents cachexia progression [3, 7]. A deeper understanding of sex‐dependent inflammatory regulation in cancer cachexia is needed.

Immune cells, particularly innate immune cells from the myeloid lineage, are the primary producers of inflammatory cytokines. Individuals with cancer cachexia consistently exhibit elevated innate immune cell content characterized by more circulating neutrophils compared to non‐cachexic cohorts [8, 9], suggesting myeloid lineage skewing. Tissue‐resident immune cell expansion has also been reported. In white adipose tissue and the gastrocnemius, macrophage content is higher during cachexia [9]. In cachectic skeletal muscle, inflammatory macrophages induce muscle wasting, while macrophage depletion decreases restores muscle cross‐sectional area (CSA) [10]. Conversely, anti‐inflammatory macrophages have a protective phenotype by preserving myotube diameter in vitro [11]. Together, these results suggest immune cell changes directly contribute to wasting; however, the precise role the immune system plays in sex‐differences observed in cachexia initiation remains unclear.

Innate immune cells are produced through myelopoiesis, whereby HSCs differentiate into multipotent progenitors (MPPs), then common myeloid progenitors (CMPs), then lineage‐restricted progenitors before terminally differentiating into granulocytes, including basophils, neutrophils, eosinophils and monocytes [12]. The HSC niche, which includes osteolineage, endothelial and adipocyte cells, controls HSC fate [13]. Under homeostasis, this regulation maintains a balance between HSC differentiation and self‐renewal, thereby preserving the HSC pool while providing sufficient replacement cells to maintain blood cell turnover [14]. Myelopoiesis can be quickly upregulated in response to signals that arise from injury, inflammation and infection, leading to myeloid skewing [13, 15]. A hallmark of cancer progression is aberrant myelopoiesis and myeloid cell recruitment into the tumour and distant tissues [16]. Haematopoietic cells migrate from the bone marrow using the CCR2‐CCL2 axis. CCR2 is expressed on the surface of monocytes, macrophages and leukocyte subsets, while CCL2 is secreted from myeloid immune cells, smooth muscle, fibroblasts and endothelial cells [17]. When CCL2 binds CCR2, migration occurs along the CCL2 gradient [18]. Cachectic muscle has higher macrophage, neutrophil and myeloid‐derived suppressor cells content [9, 10, 11], and cachectic mice have more HSCs [19]. It is unknown, however, the degree to which aberrant myelopoiesis contributes to systemic inflammation and sex‐based differences in cancer cachexia. Therefore, the objective of this study was to determine the temporal relationship between aberrant myelopoiesis, myeloid cell recruitment to skeletal muscle and cancer cachexia development in male and female mice.

## 2 Methods

Expanded descriptions of the protocols are included in the extended methods section available in Data S1.

### 2.1 Mice and Ethical Statement

Ethical approval was obtained from the University of Ottawa Animal Care and Veterinary Service Committee and conformed to the Animals for Research Act by the Canadian Council on Animal Care. Mice were housed in a pathogen‐free environment at room temperature on a 12‐h light and 12‐h dark schedule with food and water provided ad libitum until euthanasia. B6(C)‐Ccr2^tm1.1Cln^/J (Strain#027619, Jackson Laboratories) mice were bred with C57Bl/6J (Strain#000664, Jackson Laboratories) mice to produce heterozygous CCR2‐GFP mice. C57BL/6 (Strain#027, Charles River) mice from the same cohort as previously described [20] were used for all bone marrow analyses and for a subset of the body composition.

### 2.2 Study Design

At age 12 weeks, male and female mice were randomized into control or tumour‐inoculated conditions then inoculated with either 5 × 10^5^ Lewis lung carcinoma (LLC) cells in 200 μL of phosphate‐buffered solution (PBS) or 200 μL of PBS in both flanks. EchoMRI‐900 determined body composition 1 day prior to tumour inoculation and at sacrifice. Blood, bone marrow, tumour, spleen and skeletal muscle were harvested 2 (14 days), 2.5 (17 days) and 3.5 weeks (24 days) post‐inoculation in separate cohorts (Figure 1a). Mice were euthanized by CO_2_ then cervical dislocation. Body composition and CSA analysis included data from 58 mice previously reported [20].

**FIGURE 1:**
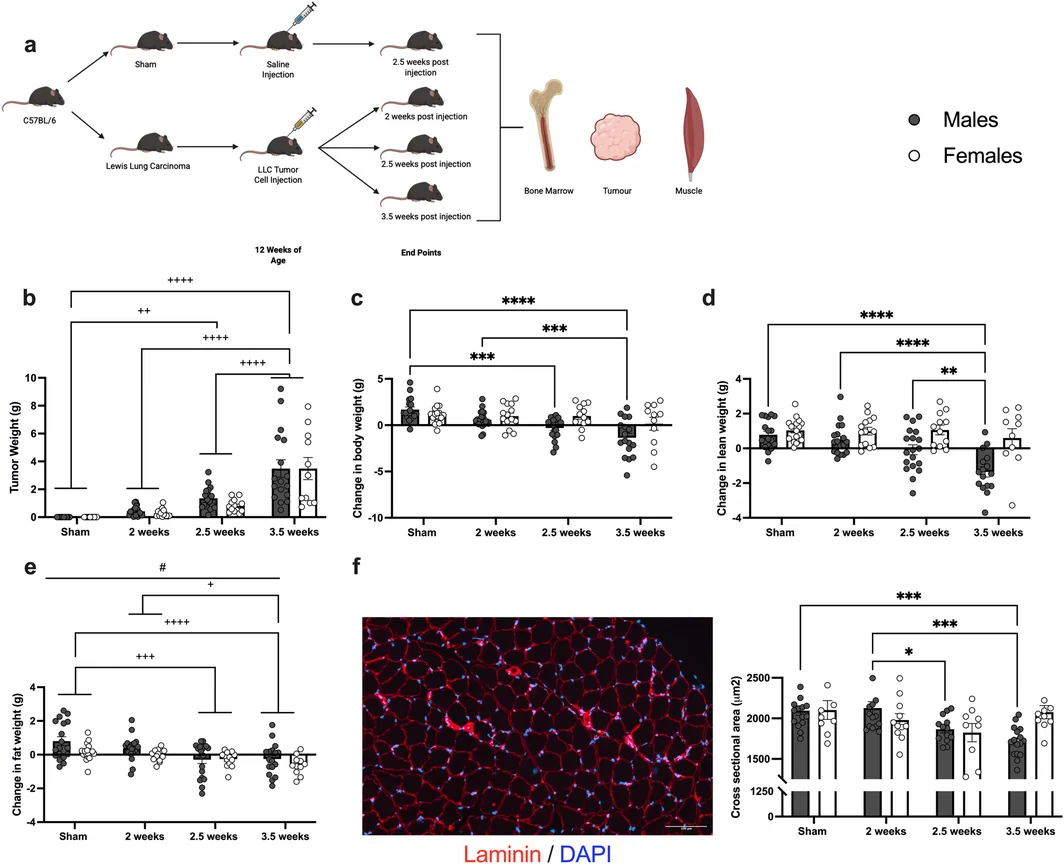
Sex‐dependent physiological and tissue changes in response to a Lewis lung carcinoma model of cachexia. (a) Study outline. Twelve‐week‐old male and female mice were randomized into either a control or cachexic group and received subcutaneous injections of PBS or Lewis lung carcinoma, respectively. Control mice were collected at 2.5 weeks post‐PBS injection, while mice within the cachexia group were randomized and collected at 2, 2.5 or 3.5 weeks post‐LLC inoculation. This image was made in Biorender. (b) Terminal tumour weight. (c) The change in tumour‐free total body weight as calculated relative to body composition at the time of injection. (d) The change in lean weight as calculated relative to body composition at the time of injection and measured by echoMRI. (e) The change in fat weight as calculated relative to body composition at the time of injection and measured by echoMRI. (f) Representative image of laminin staining (red) and gastrocnemius soleus muscle complex cross‐sectional area (CSA). Scale bar: 100 μm. *p < 0.05, **p < 0.01, ***p < 0.001, ****p < 0.0001 for sex × time interaction. ^+^ *p* < 0.05, ^++++^ *p* < 0.0001 for main effect of time. ^#^ *p* < 0.05 for main effect of sex. Two‐factor (condition and sex) ANOVA (*n* = 9–20).

### 2.3 Tissue Collection

Blood was collected immediately after sacrifice by cardiac puncture, and plasma was flash frozen and stored at −80°C. Bone marrow was collected and prepared for flow cytometry processing as previously described (extended methods) [21]. Subcutaneous tumours were resected, weighed and digested using Miltenyi tumour dissociation kit (Cat#130‐096‐730, Miltenyi Biotec) per manufacturer's instructions. Tumour homogenate was incubated in Red Blood Cell Lysing Buffer (Cat#R7767; Sigma‐Aldrich) prior to flow cytometry analysis. One gastrocnemius/soleus complex was preserved by freezing in optimal cutting temperature compound (Cat#4585, Fisher Scientific) using cooled isopentane (Cat#277258‐1L, Sigma‐Aldrich) or by flash freezing and storing at −80°C. The contralateral gastrocnemius/soleus complex was weighed and processed for flow cytometry as previously described (extended methods) [22].

### 2.4 Flow Cytometry

Samples were incubated in 100 μL of Zombie Yellow viability dye (Cat#423103, Biolegend, 1:300) for 15 min in the dark at room temperature before incubation in 200 μL of primary antibody cocktails (Tables S1–S3). Samples were washed and, where applicable, resuspended in 200 μL of streptavidin (Cat#563259, BD Bioscience, 1:800) for 30 min on ice prior to a final wash step then analysis (Attune Nxt, Thermo Fisher Scientific) with Violet (405 nm) and Blue (488 nm) excitation lasers. Gating strategies are described in Table S4, with fluorescence minus one and single‐stained controls used to establish gates (Figures [Link], [Link], [Link], [Link], [Link]). Compensation was performed using AbC Total Antibody Compensation Bead Kit (Cat#A10497, Thermo Fisher Scientific). All flow cytometry data were extracted using Attune NxT software v6.2.3.

### 2.5 Histochemical and Immunofluorescent Analysis

The mid‐belly of the gastrocnemius/soleus complex was sectioned (7 μm) using an HM525 NX cryostat and mounted on Premium Frosted Microscope Slides (Cat#12‐544‐2, Fisher Scientific). For immunofluorescent analysis, muscle cross‐sections were blocked using 5% BSA in PBS for 1 h at room temperature (RT). Sections were incubated in rat anti‐Laminin (Cat#MA1‐06100, Invitrogen, 1:200) in 5% goat serum in PBS for 2 h at RT. After several washes, sections were incubated in Alexa Fluor 594 goat anti‐rat (Cat#A‐11007, Invitrogen 1:300) in 1% BSA in PBS for 1 h at RT, and then nuclei were counterstained using 4,6‐diamidino‐2‐phenylindole (DAPI) (Cat#D9542, Sigma Aldrich). Sections were mounted and sealed with fluoromount, cover slipped and then stored in the dark at 4°C until imaging on a ZEISS CellDiscoverer 7. CSA was manually analysed with Fiji software.

### 2.6 Marrow Adipose Tissue Quantification

One tibia from each mouse was prepared and analysed as previously described (extended methods) [23].

### 2.7 C‐C Motif Chemokine Ligand 2 ELISA

CCL2 concentration was measured in plasma and muscle using the Mouse CCL2/JE/MCP‐1 Quantikine ELISA Kit (Cat#MJE00B, R and D systems) according to manufacturer's instructions (extended methods). Samples were excluded if they had a %CV ≥ 10.

### 2.8 Single‐Cell RNA‐Sequencing

We performed a secondary analysis of male‐only LLC skeletal muscle single‐cell RNA‐sequencing data [20] using RStudio (V4.40) and Seurat (V5.2.1). Sham, 2‐, 2.5‐ and 3.5‐week data were converted into individual Seurat objects and cells with > 10% mitochondrial genes, number of features ≤ 200 or ≥ 5000, or identified as doublets were removed. Gene expression was normalized using LogNormalized with a scale factor of 10 000, and the top 2000 genes were identified using FindVariable. Data were integrated using IntegrateData, and dimensionality was determined using Elbow Plots. Cell clustering was conducted using FindNeighbors and FindClusters and then visualized through a uniform manifold approximation and projection (UMAP). Cluster markers were identified through FindAllMarkers, and the subset feature was used to pool immune cell clusters. Filtration and normalization were performed for each cell population to identify subpopulations. Heat maps were generated through pheatmap and represent the average gene expression within the identified subpopulations.

### 2.9 Western Blot Analyses

Western blot analysis was performed as previously described (extended methods) [22].

### 2.10 Statistical Analyses

Data were analysed by two‐factor (condition and sex) ANOVA and Tukey's (interaction and condition) or Šidák (sex) post hoc using GraphPad Prism 9. Correlations were conducted using Pearson's R correlation test within GraphPad Prism 9, and significance between male and female correlations was determined using Fisher's R to Z transformation using the cocro package within RStudio (V4.40). Data are presented as mean ± SE, with *p* < 0.05 considered significant. All analyses were conducted by blinded investigators.

## 3 Results

### 3.1 Lewis Lung Carcinoma Selectively Induces Cachexia in Adult Male but Not Female Tumour‐Bearing Mice

To characterize sex‐specific cachexia during LLC tumour progression, we assessed tumour growth, body composition and skeletal muscle morphology across the disease time course. Tumour‐bearing adult mice exhibited progressive tumour growth regardless of sex, with tumours at 3.5 weeks significantly larger than at all other time points (Figure 1b, *p* < 0.01). At 3.5 weeks, males exhibited a significant reduction in tumour‐free body weight compared to sham and 2 weeks, and 2.5‐week tumour‐bearing males had significantly reduced tumour‐free body weight compared to sham (Figure 1c, *p* < 0.001). Conversely, females remained weight‐stable compared to sham across the time course (Figure 1c). Males also exhibited a significant reduction in lean body weight at 3.5 weeks compared to all other time points (Figure 1d, *p* < 0.01); however, females did not exhibit significant changes in lean body weight (Figure 1d). Fat weight was significantly reduced in both male and female 2.5‐ and 3.5‐week mice compared to sham (Figure 1e, *p* < 0.05). Gastrocnemius/soleus weight was significantly less in females versus males, and gastrocnemius/soleus complex weight decreased over time in both sexes (Figure S6a, *p* < 0.05). As previously reported [20], grip strength was significantly decreased at 21 and 23 days post‐inoculation regardless of sex (Figure S6b, *p* < 0.05). Myofibre CSA was significantly smaller in males at 2.5 weeks compared to 2 weeks and at 3.5 weeks compared to sham and 2 weeks with no changes in females (Figure 1f, *p* < 0.05). These data indicate that males were cachexic at 3.5 weeks, but females were not despite similar tumour growth.

### 3.2 Sex‐Specific Alterations to Haematopoiesis Precede Cachexia

To determine if altered myelopoiesis was related to sex‐differences in cachexia development, we examined if myeloid cells had altered migratory capacity via the CCR2‐CCL2 axis. Splenic weight increased regardless of sex, with spleens at 2.5 weeks significantly larger than sham and 2 weeks and spleens at 3.5 weeks significantly larger than all other time points (Figure S6c, *p* < 0.01). LLC inoculation in males led to a significant increase in HSCs at 2, 2.5 and 3.5 weeks compared to sham and in females at 2.5 weeks compared to 2 and 3.5 weeks (Figure 2a, *p* < 0.05). At 2.5 weeks, females had a significantly higher concentration of CCR2^+^‐HSCs than males (Figure 2a, *p* < 0.05). MPPs were significantly increased in males at 2, 2.5 and 3.5 weeks compared to sham, and in females at 2.5 weeks compared to sham, 2 and 3.5 weeks, with CCR2^+^‐MPPs significantly decreased in females at 3.5 weeks compared to 2 and 2.5 weeks (Figure 2b, *p* < 0.05). CMPs were significantly increased in males at 2.5 weeks compared to sham, before significantly decreasing at 3.5 weeks compared to sham, 2 and 2.5 weeks, while females had significantly decreased CMP concentration at 3.5 weeks compared to sham (Figure 2c, *p* < 0.05). CCR2^+^‐CMPs were not impacted by sex or time (Figure 2c). At 2.5 weeks, males had a significantly higher concentration of granulocyte‐monocyte progenitors (GMPs) and CCR2^+^‐GMPs compared to sham, 2 and 3.5 weeks, with CCR2^+^‐GMPs significantly increasing in females at 3.5 weeks compared to 2.5 weeks (Figure 2d, *p* < 0.05). Conversely, in males, megakaryocyte‐erythroid progenitors (MEPs) significantly increased at 2, 2.5 and 3.5 weeks compared to sham, while females exhibited significantly increased MEPs at 2.5 weeks compared to all other time points (Figure 2e, *p* < 0.05). CCR2^+^‐MEPs in females significantly increased at 2.5 weeks compared to sham, 2 weeks and 3.5 weeks (Figure 2e, *p* < 0.05). In males, long‐term HSCs (LT‐HSCs) were significantly increased at 3.5 weeks compared to all other time points, whereas in females, LT‐HSCs significantly increased at 2.5 weeks compared to 2 weeks, and CCR2^+^‐LT‐HSCs remained unchanged over time (Figure S7a, *p* < 0.05). Short‐term HSCs (ST‐HSCs) and CCR2^+^‐ST‐HSCs were unchanged by cachexia development regardless of sex (Figure S7b). No changes in common lymphoid progenitors (CLPs) and CCR2^+^‐CLPs were detected between sexes or across the timepoints (Figure S7c). These data suggest that myeloid progenitor populations undergo accelerated differentiation and migration before cachexia in males, while females have delayed CCR2^+^‐myeloid progenitor expansion.

**FIGURE 2:**
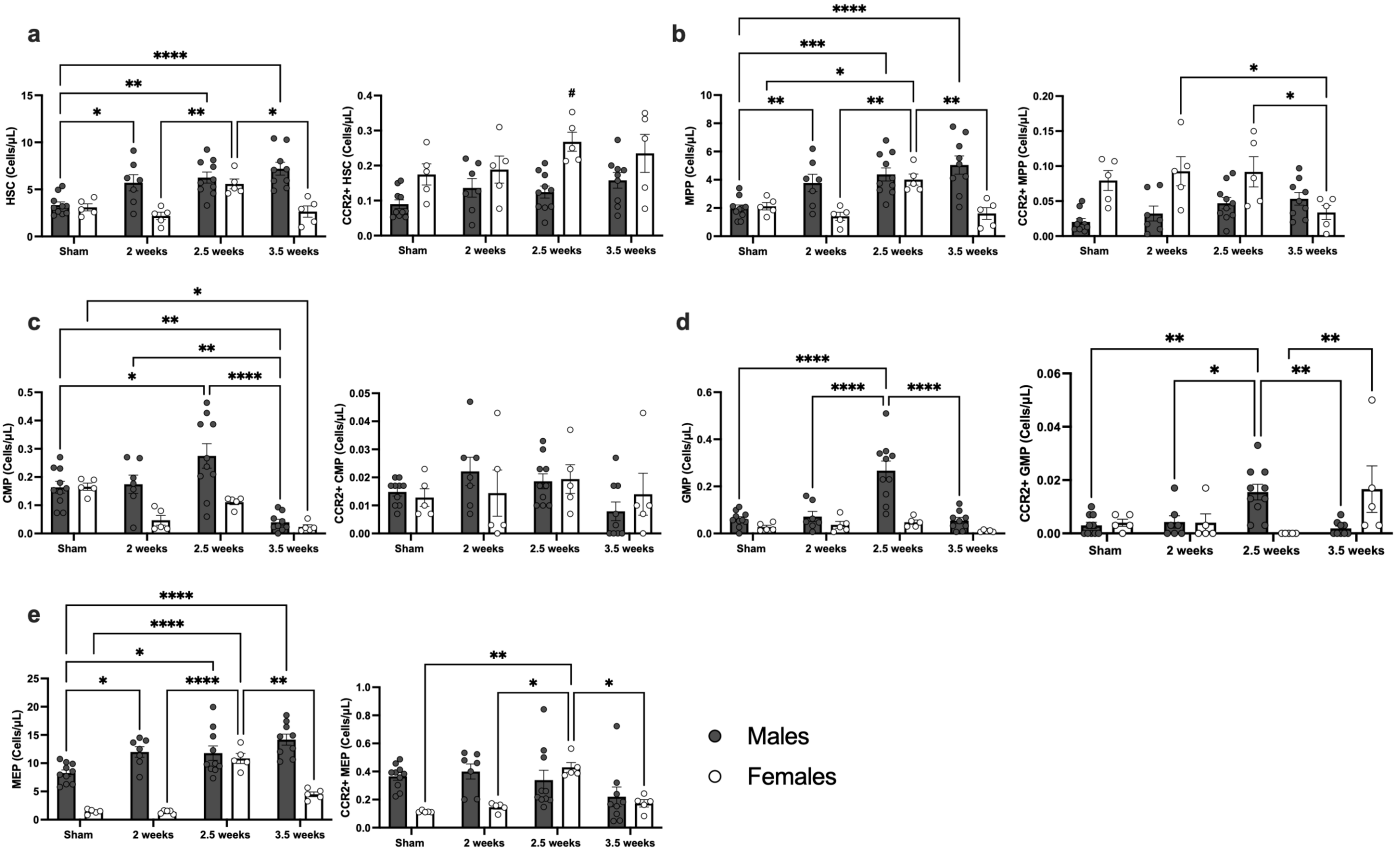
Cachexia development increases haematopoietic stem cell and progenitor cell concentrations within the bone marrow in a sex‐dependent manner. (a) Concentration of haematopoietic stem cells (HSCs) and CCR2^+^‐HSCs. (b) Concentration of multipotent progenitors (MPPs) and CCR2^+^‐MPPs. (c) Concentration of common myeloid progenitors (CMPs) and CCR2^+^‐CMPs. (d) Concentration of granulocyte‐monocyte progenitors (GMPs) and CCR2^+^‐GMPs. (e) Concentration of megakaryocyte‐erythroid progenitors (MEPs) and CCR2^+^‐MEPs. *p < 0.05, **p < 0.01, ***p < 0.001, ****p < 0.0001 for sex × time interaction. ^+^ *p* < 0.05, ^++^ *p* < 0.01, ^++++^ *p* < 0.0001 for main effect of time. ^#^ *p* < 0.05 for main effect of sex. Two‐factor (condition and sex) ANOVA (*n* = 5–10).

### 3.3 Sex‐Differences in Cellular Dynamics in the HSC Niche During Cachexia Development

To investigate whether sex‐specific haematopoietic remodelling was associated with alterations to the bone marrow niche, cellular populations involved in HSC maintenance and differentiation were assessed. In males, mesenchymal stromal cells (MSCs) were significantly elevated at 3.5 weeks compared to 2.5 weeks, and in females at 2.5 weeks compared to sham and 2 weeks (Figure 3a, *p* < 0.05). In males, adipocyte progenitors were significantly decreased at 2.5 weeks compared to 2 weeks with no changes in females (Figure 3b, *p* < 0.05). In males, osteoprogenitors were significantly increased at 2 weeks compared to sham, 2.5 and 3.5 weeks and in females at 2.5 weeks compared to sham and 3.5 weeks (Figure 3c, *p* < 0.05). In males, osteoblasts were significantly increased at 2 weeks compared to sham, 2.5‐ and 3.5‐week mice, and in females, osteoblasts were significantly lower in tumour‐bearing mice than sham (Figure 3d, *p* < 0.05). No changes in trabecular tibial marrow adipose tissue (MAT) content were detected between sexes or across the timepoints (Figure 3e). In males, endothelial progenitors were unchanged; however, in females, endothelial progenitors were significantly increased at 2.5 weeks compared to all other timepoints and at 2 and 3.5 weeks compared to sham (Figure 3f, *p* < 0.05). In males, endothelial cells were significantly increased at 2 weeks compared to 2.5 and 3.5 weeks, while in females, endothelial cells were significantly increased at 2 weeks compared to sham and 2.5 weeks (Figure 3f, *p* < 0.05). These results suggest that females have delayed HSC niche remodelling during cachexia development.

**FIGURE 3:**
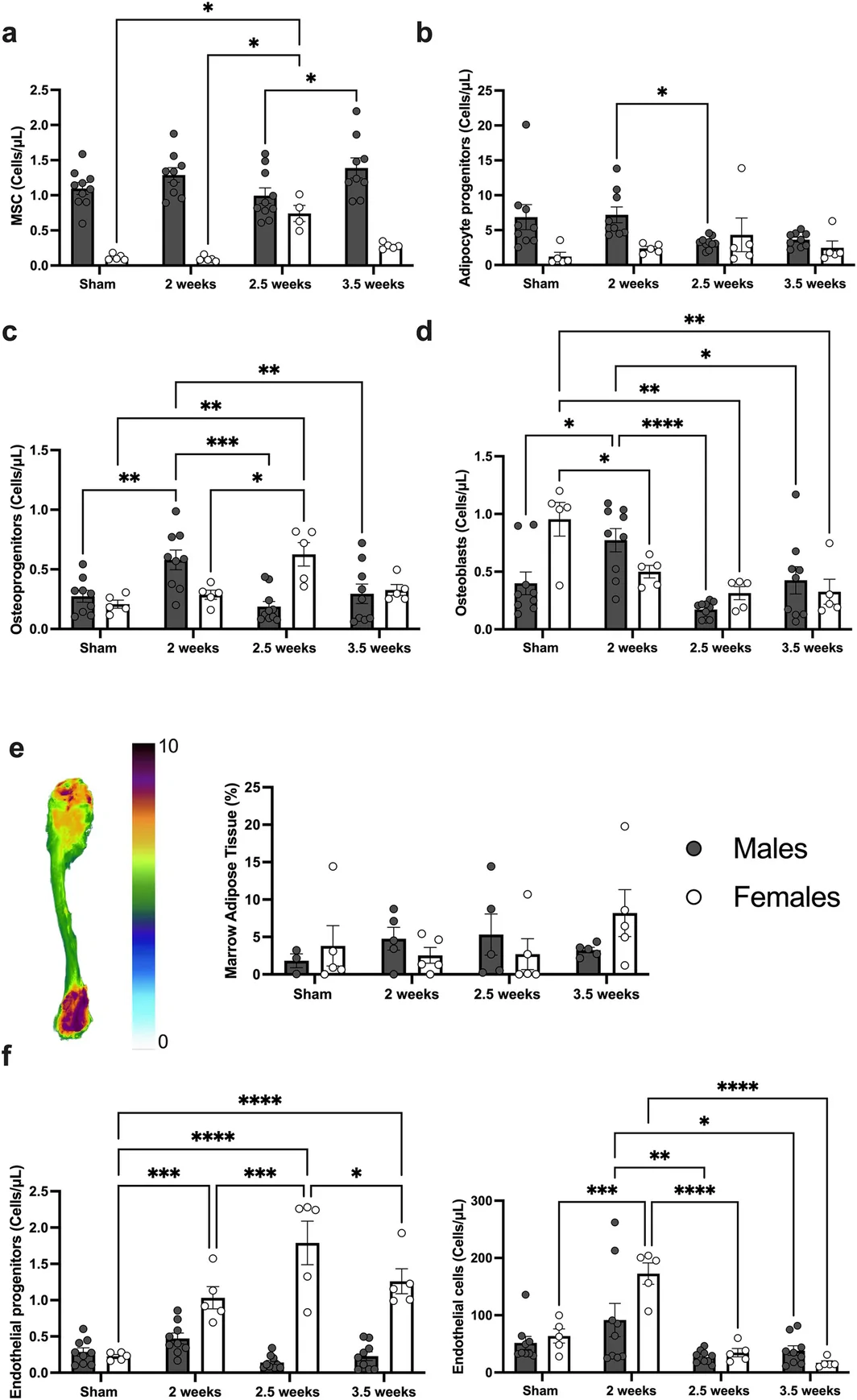
Cachexia development induces sex‐based alterations to the haematopoietic stem cell niche. (a) Concentration of mesenchymal stromal cells (MSCs). (b) Concentration of adipocyte progenitors. (c) Concentration of osteoprogenitors. (d) Concentration of osteoblasts. (e) Representative bone marrow adipose tissue (MAT) microCT images and MAT‐to‐total bone volume fraction. (f) Concentration of endothelial progenitors and endothelial cells. *p < 0.05, **p < 0.01, ***p < 0.001, ****p < 0.0001 for sex × time interaction. Two‐factor (condition and sex) ANOVA (*n* = 3–10).

### 3.4 Cachexia Development Reduces Bone Marrow‐Derived Immune Cell Migration to the Tumour

To examine if the CCL2‐CCR2 axis was involved in tumorigenesis, we quantified CCL2 and immune cells expressing CCR2 within tumours. Plasma CCL2 concentration significantly increased at 2.5 weeks compared to sham and at 3.5 weeks compared to all other timepoints regardless of sex (Figure 4a, *p* < 0.01). CCR2^+^‐HSCs were significantly decreased at 3.5 weeks compared to 2 weeks, but CCR2^+^‐MPPs were not changed regardless of sex (Figure 4b, *p* < 0.05). CCR2^+^‐CMP and CCR2^+^‐GMP were unchanged throughout the time course; however, CCR2^+^‐MEPs were significantly decreased at 3.5 weeks compared to 2.5 weeks regardless of sex (Figure 4c, *p* < 0.01). CCR2^+^‐macrophages were significantly decreased at 3.5 weeks compared to 2 weeks, while CCR2^+^‐CD206^−^ macrophages were unchanged, and CD206^+^ macrophages were significantly decreased at 3.5 and 2.5 weeks compared to 2 weeks regardless of sex (Figure 4d, *p* < 0.05). CCR2^+^‐LT‐HSCs were significantly decreased at 3.5 weeks compared to 2 weeks regardless of sex (Figure S8a, *p* < 0.05). CCR2^+^‐ST‐HSCs were significantly decreased at 2.5 and 3.5 weeks compared to 2 weeks, with females having a significantly higher concentration than males at 2 weeks (Figure S8b, *p* < 0.01). CCR2^+^‐CLPs were unchanged throughout tumour growth regardless of sex (Figure S8c). These results indicate that although plasma CCL2 levels increase with tumour growth, bone marrow‐derived cells do not accumulate in the tumour.

**FIGURE 4:**
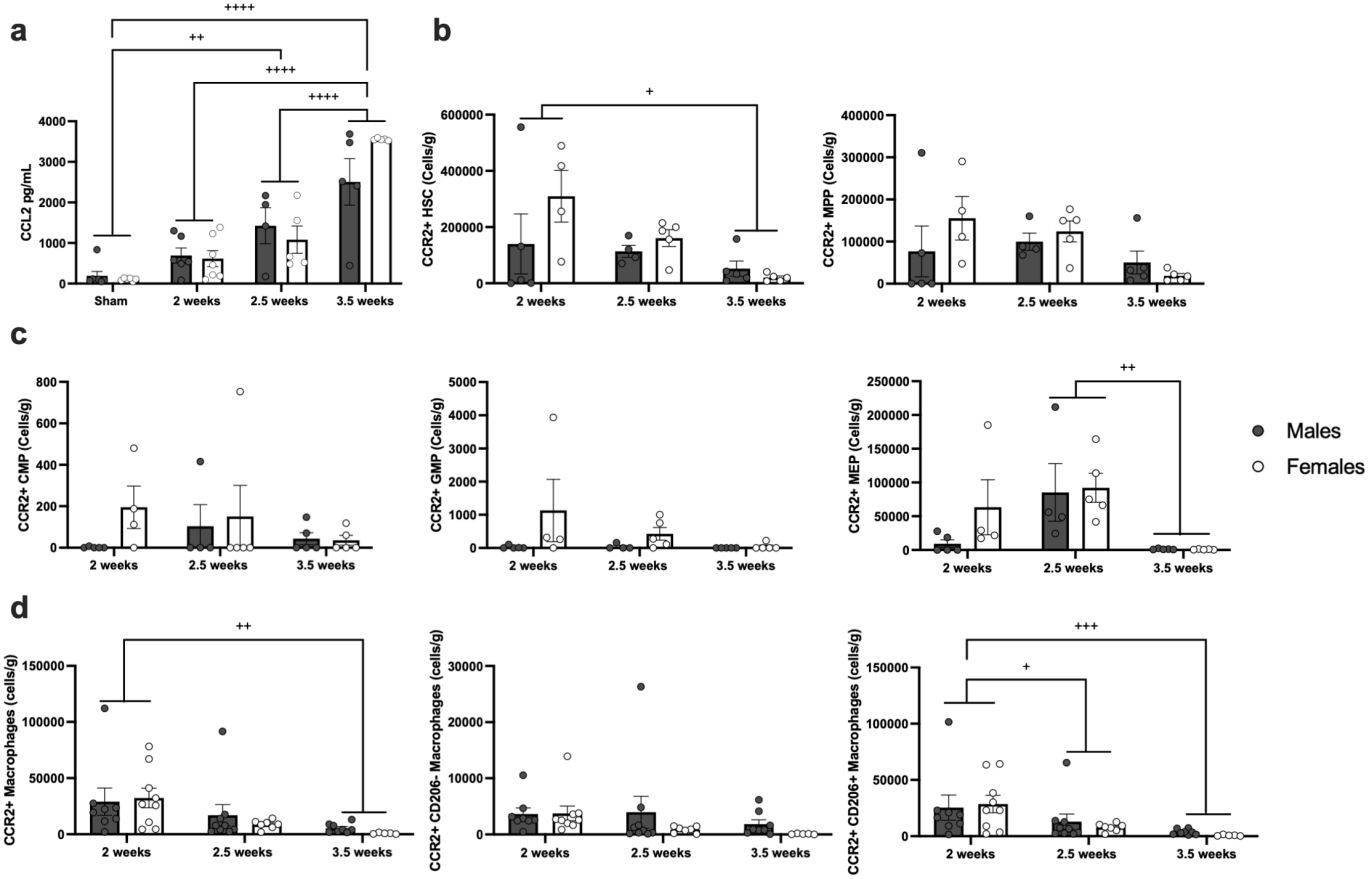
Bone marrow derived immature and mature immune cells within the tumour decrease with tumour growth and cachexia development. (a) Concentration of CCL2 in the plasma. (b) CCR2^+^‐HSCs per gram of tumour. HSCs and MPPs. (c) CCR2^+^‐HSPCs per gram of tumour. CMPs, GMPs and MEPs. (d) CCR2^+^‐macrophages per gram of tumour. F4/80^+^ cells, F4/80^+^ CD206^−^ cells and F4/80^+^ CD206^+^ cells. ^+^ *p* < 0.05, ^++^ *p* < 0.01, ^+++^ *p* < 0.001, ^++++^ *p* < 0.0001 for main effect of time. Two‐factor (condition and sex) ANOVA (*n* = 4–9).

### 3.5 In Males, Myeloid Cells Markers and CCL2‐CCR2 Signalling Markers Accumulate During Cachexia Development

Given the reduced accumulation of CCR2^+^‐haematopoietic populations in the tumour despite elevated circulating CCL2, we next investigated whether skeletal muscle represented an alternative site by reanalysing our male‐only scRNAseq dataset [20]. Reclustering the muscle‐resident immune cell population, we identified seven unique cell clusters based on canonical gene expression (Figure 5a). Tumour‐bearing mice had proportionally more neutrophils and monocytes and proportionally fewer lymphocytes compared to sham (Figure 5b, Table S5). *Ccr2* expression in monocytes decreased at 2.5 weeks compared to all other time points (Figure 5c, Table S6). *Ccl2* expression increased with cachexia development in all cell clusters. At 2 weeks, *Ccl2* expression is predominantly macrophage driven, while at 3.5 weeks, *Ccl2* is expressed in a variety of immune and non‐immune cell populations (Figure 5d, Table S7). These results suggest that within male mice, macrophages drive early immune cell recruitment to the muscle, with several cell types contributing later.

**FIGURE 5:**
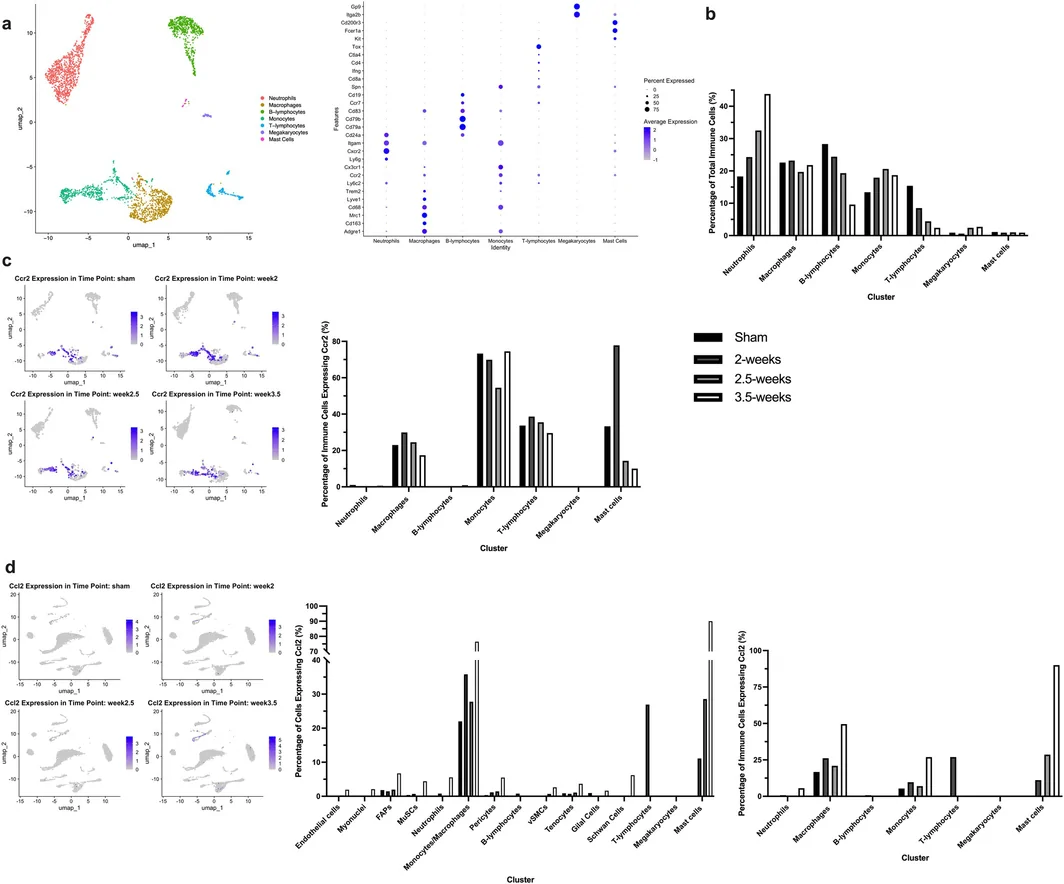
In males, myeloid cells markers and CCL2‐CCR2 signalling markers accumulate during cachexia development. (a) Uniform manifold approximation and projection (UMAP) clustering of immune subclusters and feature plot of different markers identifying cell types. (b) Relative cell percentage (%) of immune cell subclusters. (c) Expression of *Ccr2* within immune cell subclusters. Uniform manifold approximation and projection (UMAP) representing the expression of *Ccr2* and the relative percentage of cells expressing *Ccr2*. (d) Expression of *Ccl2* within immune cell subclusters. Uniform manifold approximation and projection (UMAP) representing the expression of *Ccl2* and the relative percentage of cells expressing *Ccl2*.

### 3.6 Cachexia Development Promotes Bone Marrow‐Derived Macrophage Accumulation Within Skeletal Muscle With Sex‐Dependent Effects on Skeletal Muscle Cross‐Sectional Area

Given the increased myeloid immune cell content and elevated CCL2‐CCR2 signalling identified within males by scRNA‐seq, we quantified CCL2 and immune cells expressing CCR2 in skeletal muscle throughout tumour progression in both males and females. CCL2 concentration significantly increased at 3.5 weeks compared to sham and 2 weeks regardless of sex (Figure 6a, *p* < 0.05). Neutrophils were significantly increased at 3.5 weeks compared to sham regardless of sex (Figure 6b, *p* < 0.01). At 2.5 weeks, resident/persistent Ly6C^low^CCR2^low^CX3CR1^hi^ monocyte/macrophages were significantly increased compared to 2 weeks regardless of sex (Figure 6c, *p* < 0.01). Macrophage proportions remained unchanged across the time course, but males had a significantly higher macrophage proportion at sham and 2.5 weeks than females (Figure 6d, *p* < 0.05). CD206^−^ macrophages were proportionally more abundant in females, while CD206^+^ macrophages were proportionally more abundant in males (Figure 6d, *p* < 0.05). CCR2^+^‐macrophage proportions were increased at 2.5 weeks compared to sham and at 3.5 weeks compared to sham and 2 weeks, due to increased inflammatory CCR2^+^CD206^−^ macrophages at 2.5 and 3.5 weeks compared to sham regardless of sex (Figure 6e, *p* < 0.05). Males had proportionally more CCR2^+^CD206^+^ macrophages regardless of time point than females (Figure 6e, *p* < 0.05). We next compared correlations between these immune cell populations and muscle atrophy between males and females to understand sex‐differences in how these cell populations related to cachexia. In males, a weak, non‐significant correlation was observed between neutrophils and CSA; however, in females, there is a moderate negative correlation (Figure 6f, *r* = −0.196, *p* < 0.05). Males have a moderate, significant positive correlation (Figure 6f, *r* = 0.584, *p* < 0.01), while females have a non‐significant, moderate negative correlation between Ly6C^hi^CCR2^+^CX3CR1^low^ monocytes and CSA. In males, CCR2^+^‐macrophages have a non‐significant, moderate negative correlation with CSA, which is significantly different (Figure 6f, *p* < 0.05) from the non‐significant, moderate positive correlation observed within females. All other cells have no significant correlations with CSA and non‐significant differences in correlations between sexes (Figure S9). These results suggest that sex‐differences in immune subsets relate to cachexia development in males.

**FIGURE 6:**
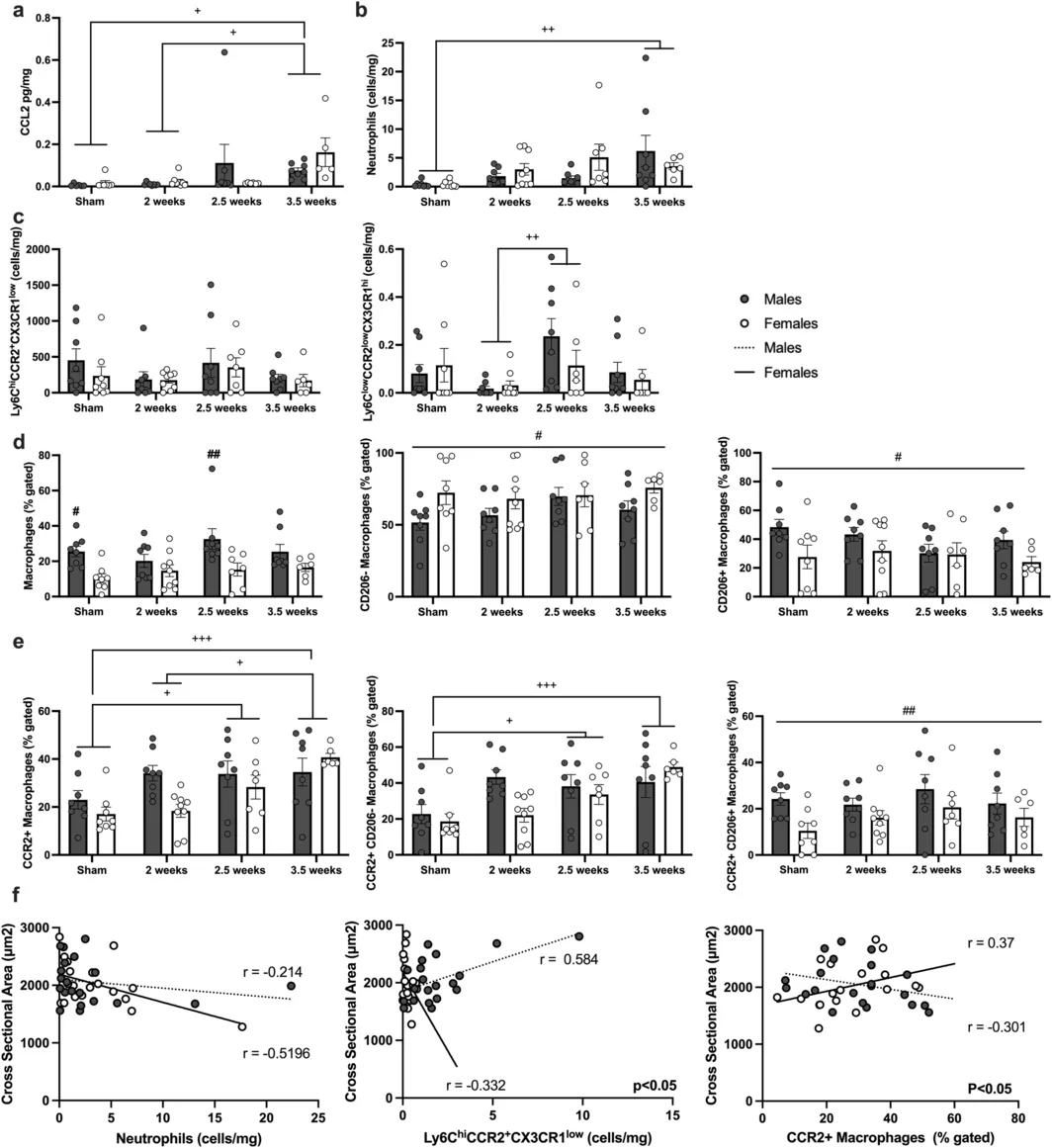
Cachexia development promotes bone marrow‐derived macrophage accumulation within skeletal muscle with sex‐dependent effects on skeletal muscle cross‐sectional area. (a) Concentration of CCL2 per milligram of gastrocnemius soleus complex muscle tissue. (b) Neutrophils per milligram of muscle. Ly6G^+^ cells. (c) Monocytes per milligram of muscle. Ly6C^hi^CCR2^+^CX3CR1^low^ cells and Ly6C^low^CCR2^low^CX3CR1^hi^ cells. (d) Proportion of macrophages. F4/80^+^ cells, F4/80^+^CD206^−^ cells and F4/80^+^CD206^+^ cells. (e) Proportion of CCR2^+^‐macrophages. F4/80^+^ cells, F4/80^+^CD206^−^ cells and F4/80^+^CD206^+^ cells. (f) Correlation between neutrophils, Ly6C^hi^CCR2^+^CX3CR1^low^ cells, CCR2^+^‐macrophages and cross‐sectional area. ^+^ *p* < 0.05, ^++^ *p* < 0.01, ^+++^ *p* < 0.001 for the main effect of time. ^#^ *p* < 0.05, ^##^ *p* < 0.01 for main effect of sex. Two‐factor (condition and sex) ANOVA, Pearson's correlation test and Fisher's R to Z transformation (*n* = 5–9).

### 3.7 Macrophages Transcribe Atrophic Factors With Corresponding Changes to Myonuclei Transcription Prior to Cachexia Development

To further connect sex‐differences in monocytes/macrophages and CSA, we next examined atrophic signalling pathways. Our scRNA‐seq data revealed that in males, macrophages have higher expression of the atrophic factors: *Il‐1*, *Tgfb1*, *Tnfsf12* and *Tnf*, at 2 and 3.5 weeks, while monocytes and neutrophils upregulate these factors only at 3.5 weeks (Figure 7a). Myonuclei upregulate the corresponding receptors, *Il1r1*, *Tgfbr1*, *Tnfrsf1a* and *Tnfrsf12a*, at 2 and 3.5 weeks along with signalling factors, *Col1a1* at 2 weeks, *Serpine1* at 2.5 weeks and *Smad3* and *Foxo1* at 2 and 2.5 weeks with a subsequent upregulation of atrogenes, *Trim63* and *Fbxo32*, at 3.5 weeks (Figure 7a). These transcriptomic data suggest that within males, macrophages initiate atrophic signalling prior to cachexia development. However, SMAD3, P‐SMAD3, SMAD7 and TGFBR1 protein were unchanged throughout cachexia development regardless of sex in muscle homogenate (Figure 7c–e).

**FIGURE 7:**
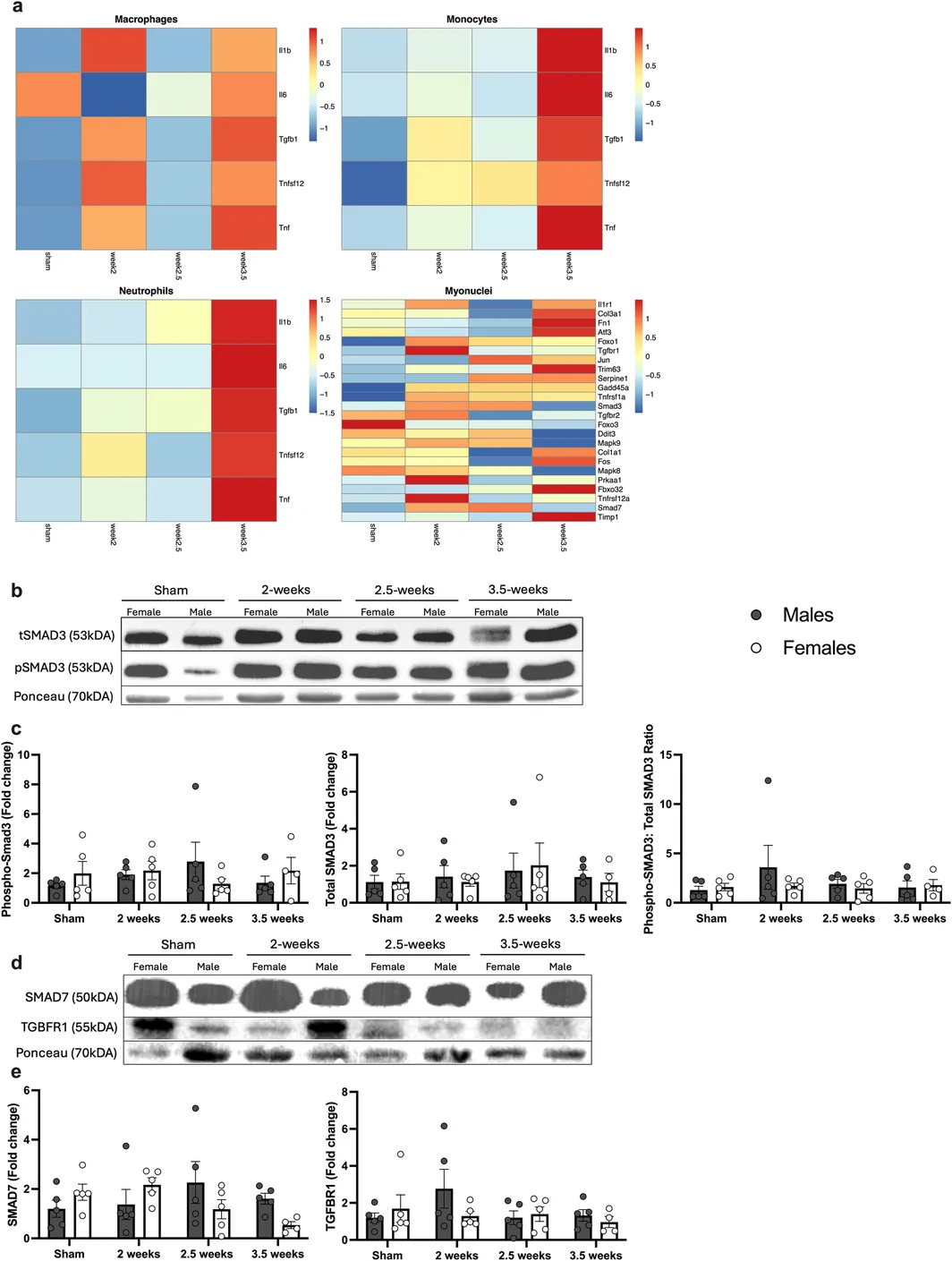
Macrophages transcribe atrophic factors with corresponding changes to myonuclei transcription prior to cachexia development. (a) Heat maps of the expression of pro‐atrophic factors in macrophages, monocytes, neutrophils and myonuclei within male skeletal muscle. (b) Representative western blot of whole skeletal muscle of SMAD‐3 and pSMAD3. (c) Quantification of SMAD‐3 and pSMAD‐3 protein expression. (d) Representative western blot of whole skeletal muscle of SMAD7 and TGFBR1. Two‐factor (condition and sex) ANOVA (*n* = 4–5).

## 4 Discussion

Inflammation is a hallmark of cachexia [4, 8, 9], which can be traced back to the bone marrow through alterations in HSC content and fate [19]. In the present study, we sought to determine the temporal relationship between aberrant myelopoiesis, HSC niche dysregulation, myeloid cell recruitment to skeletal muscle and cancer cachexia development in a sex‐dependent manner. Our results demonstrate that aberrant myelopoiesis occurs in males prior to cachexia development. After an initial increase in tumour myeloid cell content, marrow‐derived myeloid cells in the tumour are relatively decreased, suggesting that continued overproduction of myeloid cells does not sustain tumour growth. Rather, myeloid cell accumulation in skeletal muscle coincides with increased CCL2 levels in both males and females, with sex‐differences detected in certain subpopulations with myeloid populations generally related to decreases in CSA in males. Together, these results suggest that aberrant myelopoiesis and HSC niche remodelling are early events in cancer cachexia within males and that differences in myeloid lineage cell composition in skeletal muscle may underlie sex‐differences in cachexia.

Aberrant myelopoiesis has been observed in several disease states with tumour‐derived factors inducing aberrant myelopoiesis in cancer [24]. Immunosuppressive CCR2^+^‐monocytes and macrophages infiltrate tumours during the immune evasion phase, which enables tumour growth, and CCR2 depletion inhibits metastasis, increases survival and prevents cancer recurrence [25, 26, 27]. Myelopoiesis is initiated when inflammatory cytokines such as interferon type 1 and 2, IL‐1 and TNF‐α activate HSCs and transcriptionally prime them towards a myeloid fate [15]. Concurrently, GMPs accelerate differentiation, resulting in mature myeloid cell populations expansion [28]. These results align with our study, where males undergo HSC expansion prior to an increase in CMPs and GMPs, along with an increase in CCR2^+^‐GMPs, suggesting aberrant myelopoiesis and CCR2^+^‐myeloid progenitor expansion prior to cachexia onset in males. Conversely, females undergo timepoint‐specific remodelling and delayed CCR2^+^‐myeloid progenitor expansion, without cachexia onset. Since sex‐differences in the tumour myeloid component were not detected, with myeloid lineage cells decreasing as tumours progressed, these results suggest that the persistent myelopoiesis may be related to other systemic pathologies associated with advanced cancer. Our findings extend previous literature demonstrating sex‐based differences in haematopoiesis at the cellular [26, 27] and molecular level [29]. The early and specific aberrant myelopoiesis in males suggests that sex‐dependent differences in haematopoiesis in response to tumours may limit myeloid‐driven inflammation in females, thereby preventing muscle wasting.

Cellular components of the HSC niche regulate HSC maintenance, activation and differentiation. Osteoblast concentration is correlated to HSC concentration, with activated HSCs localized to active bone remodelling sites [30, 31]. Osteolineage cells, particularly osteoprogenitors, secrete haematopoietic supportive growth factors, causing HSC expansion [30, 32]. Studies further suggest that osteoblasts can provide specialized niches for lymphoid progenitors [32]. In males, osteoprogenitors increased at 2 weeks, which coincides with the increase in HSCs, suggesting that osteoprogenitors support HSC expansion in cancer cachexia. With osteolineage cells, endothelial cells contribute to HSC maintenance and activation through a similar complement of secreted paracrine factors [32]. Females had delayed osteoprogenitor expansion, which correlates with their timepoint‐specific HSC and myeloid progenitor expansion and occurred after the endothelial cell elevation at 2 weeks. This temporal sequence may have contributed to the myeloid progenitor expansion at 2.5 weeks. Furthermore, females had elevated endothelial progenitors within tumour‐bearing mice, which may favour HSC maintenance and be associated with delayed HSC expansion. Marrow adipocytes provide energy during emergency myelopoiesis to fuel HSC differentiation as directed by other niche cells [33]. Excess MAT negatively impacts haematopoiesis by decreasing the available space and prematurely exhausting HSCs [13]. Adipocyte progenitors decrease at 2.5 weeks in males; however, MAT remains unchanged. Given that MSCs within the bone marrow are skewed towards adipogenic fate in cachexia [19], our results may reflect altered MAT turnover, thereby preventing MAT accumulation. Overall, sex‐specific HSC niche remodelling likely contributes to the sex‐difference in haematopoiesis observed in cachexia and may contribute to the difference in cachexia prevalence between males and females.

Our results show that CCL2 levels increased in 3.5‐week muscle, and the proportion of CCR2^+^‐macrophages, particularly the inflammatory CD206^−^ population, within muscle increased prior to cachexia onset. Interestingly, we also observed that early infiltrating monocytes (Ly6C^hi^CCR2^hi^CX3CR1^low^) [34, 35] were not changed at our timepoints, but persistent monocyte/macrophages (Ly6C^low^CCR2^low^CX3CR1^high^) [34, 35] were elevated at 2.5 weeks in males and females. Our time course provides resolution on monocyte/macrophage changes within cachectic skeletal muscle and suggests that early peripheral monocyte infiltration leads to an increase in persistent monocytes with inflammatory/repair properties. Further, the proportion of CCR2^+^‐macrophages within muscle increased in both sexes while the overall macrophage concentration did not change, indicating that bone marrow‐derived macrophages become the primary contributors to the muscle macrophage pool. The observed CCR2^+^‐macrophage accumulation aligns with literature indicating that macrophage/CCR2 depletion attenuates muscle wasting [10, 11]. The lack of an increase in the overall macrophage population differs from past literature that reported increases in macrophage content [10, 11]. This discrepancy may be due to experimental design both previous studies seeded cancer cells directly into muscle and pancreas, respectively [10, 11]. Intramuscular tumour seeding causes the tumour and muscle microenvironment to become interwoven, making it difficult to determine the source of immune cell changes. Cachexia also varies with tumour inoculation site, and seeding tumour cells directly into the pancreas induces more severe cachexia than subcutaneous tumour seeding, which likely elicits different immunological responses [36].

Although macrophage accumulation was similar in males and females, we did detect sex‐differences in macrophage subsets, which may explain sex‐differences in the macrophage‐to‐cachexia relationship. Male muscle was enriched for CD206^+^ ‘M2’ macrophages, and female muscle was enriched for CD206^−^ ‘M1’ macrophages. The relative abundance of CD206^+^ ‘M2’ macrophages in males compared to females was primarily due to increases in the CCR2^+^‐subset. These results were further supported by the opposing correlation between CCR2^+^‐macrophages and CSA, indicating that CCR2^+^‐macrophage subpopulations may have opposite functions in skeletal muscle wasting based on sex. This assertion is supported by Pryce et al. [11] who similarly reported a dual role for macrophages to induce and protect against cancer cachexia depending on their polarization, thus highlighting their dynamic role in muscle maintenance and repair. Together, our findings reveal that aberrant myelopoiesis coincides with increased CCR2^+^‐macrophage recruitment to the muscle, thereby maintaining the local macrophage pool, with sex‐differences in macrophage subpopulations likely contributing to cachexia development in males.

Elevated levels of CCL2 inhibit myoblast and myotube function and induce skeletal muscle wasting through SMAD3‐, JNK‐ and AMPK‐dependent signalling in tumour and non‐tumour models [37]. Given the observed rise in muscle CCL2 levels and CCR2^+^‐macrophage accumulation, we sought to understand how these signalling pathways are activated across our time course. Although we did not directly test a specific mechanism, scRNAseq analysis of male muscle reveals that macrophages upregulate atrophic ligands prior to cachexia initiation compared to neutrophils and monocytes that upregulate these ligands during cachexia. Myonuclei upregulated receptors for these ligands prior to cachexia development, followed by expression of genes associated with muscle protein breakdown during cachexia, thereby suggesting that macrophages produce ligands to initiate cachexia development prior to other immune cells. Conversely, protein‐level analysis of these atrophic receptors and signalling factors in whole muscle homogenates did not reveal any changes across the cachexia time course regardless of condition or sex. The differences between the transcription and protein level date maybe is not surprising given the potential for fibre‐type specific changes in cachexia [38], post‐translational regulation and sensitivity and cellular specificity of the transcriptomic results. A limitation of the work was not being able to mechanistically link this pathway to muscle wasting; however, future research should examine the identified signalling pathways in a cell type‐specific manner.

Together, our results suggest that aberrant myelopoiesis is an early occurrence in cancer cachexia, which is not solely to support tumour growth. Sex‐specific HSC niche remodelling and early aberrant myelopoiesis in males precede cachexia development. Marrow‐derived macrophage accumulation occurs in both males and females; however, differences in macrophage composition may underlie sex‐based differences in cancer cachexia prevalence. Although the present study did not directly investigate mechanisms linking aberrant myelopoiesis to cancer‐induced muscle wasting, our scRNA‐seq results suggested that, in males, muscle macrophages initiate atrophic signalling in myonuclei prior to overt muscle wasting. Directly comparing males and females across several outcomes throughout our time course allowed for novel insights into sex‐specific mechanisms driving cachexia, representing an important first step in understanding the role aberrant myelopoiesis plays in cancer cachexia and establishing it as a potential therapeutic target.

## Funding

This work was supported by a CIHR Project grant to M.D.L. and N.W‐B. A.T. was supported by a Canadian Institute of Health Research (CIHR) Graduate Scholarship (Master's). A.B. and L.E.O. were supported by the Ontario Graduate Scholarship (OGS). J.V. was supported by the uOttawa/CHEO Research Institute's Doctoral Fellowship for Advancement of Biological Perspectives for Exercise Interventions Across the Lifespan and OGS. N.C. was supported by the Chilean National Agency for Research and Development, the uOttawa/CHEO Research Institute's Doctoral Fellowship for Advancement of Biological Perspectives for Exercise Interventions Across the Lifespan, and the uOttawa Eric Poulin Centre for Neuromuscular Disease Scholarship in Translational Research and the uOttawa Brain and Mind Research Institute. C.B. was supported by a CIHR Graduate Scholarship (Master's).

## Conflicts of Interest

The authors declare no conflicts of interest.

## Supporting information


**Table S1:** Bone marrow flow cytometry primary antibody cocktails.
**Table S2:** Tumour flow cytometry primary antibody cocktails.
**Table S3:** Muscle flow cytometry primary antibody cocktails.
**Table S4:** Bone marrow‐derived cell flow cytometry gating strategies.
**Table S5:** Percent of immune cells within skeletal muscle across the experimental time course.
**Table S6:** Percent of immune cells within skeletal muscle that express CCR2 across the experimental time course.
**Table S7:** Percent of cells within skeletal muscle that express CCL2 across the experimental time course.

**Data S1:** Supporting information.

**Figure S1:** Flow cytometry gating strategies for haematopoietic stem cells.

**Figure S2:** Flow cytometry gating strategies for myeloid progenitors.

**Figure S3:** Flow cytometry gating strategies for mesenchymal stromal cells.

**Figure S4:** Flow cytometry gating strategies for haematopoietic stem cell niche cells.

**Figure S5:** Flow cytometry gating strategies for mature myeloid cells.

**Figure S6:** Changes to muscle weight, grip strength, and splenic weight following LLC Inoculation. (a) Terminal gastrocnemius soleus complex weight. (b) Grip strength across the experimental time course in male and female mice. (c) Terminal spleen weight. ^++^ *p* < 0.01, ^+++^ *p* < 0.001, ^++++^ *p* < 0.0001 for main effect of time. ^##^ *p* < 0.01, ^###^ *p* < 0.001, ^####^ *p* < 0.0001 for main effect of sex. A represents a significant difference (*p* < 0.05) from days −1, 10 and 14. B represents a significant difference (*p* < 0.05) from days −1, 7, 10, 14 and 17. Two‐factor (condition and sex) ANOVA or two‐factor (time and sex) ANOVA (*n* = 5–20).

**Figure S7:** Changes to LT‐HSCs, ST‐HSCs and CLPs within the bone marrow following LLC inoculation. (a) Concentration of LT‐HSC and CCR2^+^‐LT‐HSC. (b) Concentration of ST‐HSC and CCR2^+^‐ST‐HSCs. (c) Concentration of CLP and CCR2^+^‐CLPS. **p < 0.01, ***p < 0.001, ****p < 0.0001 for sex × time interaction. ^#^ *p* < 0.05 for main effect of sex. Two‐factor (condition and sex) ANOVA (*n* = 5–10).

**Figure S8:** Changes to LT‐HSCs, ST‐HSCs and CLPs within the tumour following LLC inoculation. (a) Concentration of LT‐HSC and CCR2^+^‐LT‐HSCs. (b) Concentration of ST‐HSC and CCR2^+^‐ST‐HSCs. (c) Concentration of CLP and CCR2^+^‐CLPS. ^+^ *p* < 0.05, ^++^ *p* < 0.01 for main effect of time. ^#^ *p* < 0.05 for main effect of sex. Two‐factor (condition and sex) ANOVA (*n* = 4–9).

**Figure S9:** Relationships between immune cells within skeletal muscle and cross‐sectional area. (a) Correlation between monocytes and cross‐sectional area. Ly6C^low^CCR2^low^CX3CR1^hi^ cells. (b) Correlation between macrophages and cross‐sectional area. F4/80^+^ cells, F4/80^+^CD206^−^ cells and F4/80^+^CD206^+^ cells. (c) Correlation between CCR2^+^‐macrophages and cross‐sectional area. F4/80^+^CD206^−^ cells and F4/80^+^CD206^+^ cells. Pearson's correlation test and Fisher's R to Z transformation (*n* = 5–9).

## Data Availability

The data that support the findings of this study are openly available in Cachexia Muscle Time course at https://github.com/musclesci/CachexiaMuscleTimecourse.git.
