# Supplementary material for: Aberrant Myelopoiesis and Haematopoietic Stem Cell Niche Remodelling Precedes Cancer Cachexia Onset in a Sex‐Dependent Manner
Source: J Cachexia Sarcopenia Muscle. 2026 Oct 16;17(5):e70403. doi: 10.1002/jcsm.70403 (PMC13673406; doi:10.1002/jcsm.70403)
Supplement: Supplementary file 1 [file JCSM-17-e70403-s004.docx]

**Supplemental Methods**

*Bone marrow isolation*

Femurs were resected, and metaphysis were cut using surgical scissors. Bone marrow was flushed from the marrow cavities with 1 mL of 5% fetal bovine serum (FBS) in PBS using a 25-gauge needle. Samples were then centrifuged at 500g for 5 minutes at 4°C, and the supernatant removed, while the cell pellet was resuspended in 1 mL of 5% FBS and left on ice for further processing. The femurs were then carefully crushed using a mortar and pestle and chopped in 3mL of 0.2% type 2 collagenase diluted in high glucose Dulbecco’s Modified Eagle Medium (DMEM). The samples were then incubated at 37°C and 5% CO2 for 45 minutes with agitation every 15 minutes. Samples were then mixed with remaining bone marrow cells before being filtered through a 70µm filter. The cell suspension was centrifuged at 500g for 5 minutes at 4°C. The samples were then divided for immediate processing for flow cytometry or flash frozen and stored at -80°C. Samples aliquoted for flow cytometry were then processed as described within the flow cytometry analysis section.

*Muscle preparation for flow cytometry*

Muscles were transferred to a Miltenyi GentleMACS C tube (Cat#130-093-237, Miltenyi Biotec) containing Collagenase B (1.5U/ml, Cat#11088831001; Roche® Life Science Products,) and Dispase II (2U/ml, Cat#42613-33-2; Sigma Aldrich) in Ham’s F10 media (Cat#318-050-CL, Wisent Inc, St-Bruno, Canada). Samples were homogenised in the gentleMACS Octo Dissociator (Cat#130-096-427, Miltenyi Biotec). Muscle homogenate was filtered through a 100µm and a 70µm filter, treated with 1mL of red Blood Cell Lysing Buffer (Hydri-MaxTM; Cat#R7767; Sigma-Aldrich) for 3 minutes on ice then divided for antibody staining. Samples were quenched in 10mL of PBS and centrifuged at 400g for 5 minutes at 4°C. Cell pellets were resuspended in 200µL of PBS and split into two centrifuged at 400g for 5 minutes at 4°C and processed as described within the flow cytometry analysis section.

*Marrow adipose tissue quantification*

One tibia from each mouse was fixed in 10% formalin for 48 hours and decalcified in 14% EDTA (pH 7.4) for 14 days. Decalcified bones were stored in 70% ETOH at 4°C until stained with osmium tetroxide. Bones were placed in 1mL of 1% osmium tetroxide solution, made of a 1:1 dilution of 2% osmium tetroxide and 5% potassium dichromate in distilled water, for 48 hours. The bones were washed with running cold water for 1 hour and stained a second time with 1mL of 1% osmium tetroxide solution for 48 hours. After a second wash, bones were stored in 70% ETOH at 4°C until imaging. µCT imaging was conducted using a Bruker Skyscan 1272 (Bruker, Kontich, Belgium) with 70kV, 142mA, 0.5mm A1 ﬁlter, 550ms integration time, 1° rotation step, 360 angular range, and 12mm image pixel size. Bone images were reconstructed using NRecon and analysed using CTan (Bruker, Kontich, Belgium) with a 100 global threshold value. Total marrow adipose tissue was quantiﬁed as a percentage of total bone volume.

*Muscle Protein Extraction*

For ELISA, flash frozen muscle was homogenised in RIPA buffer (10µl/mg) containing Complete Protease inhibitor cocktail (Cat#04693116001, Millipore Sigma) and Phosphatase inhibitor (Cat#4906837001, Millipore Sigma) using a bead homogeniser (Fisher Scientific Bead Mill 24, Thermo Fisher Scientific) for two 1-minute cycles at 6m/s, separated by 5s. Homogenised samples were centrifuged at 1500g for 15 minutes at 4°C and stored in -80°C until ELISA was performed.

*Western blot analysis*

Protein content within whole muscle lysate was quantified using Bradford assay (Cat# 5000006, Bio-Rad) following the manufacturer's instructions and sample protein concentration was normalized using 4x Laemmli buffer. Protein extracts where subjected to SDS-PAGE. To account for inter-gel variability, a standard lane was loaded with a pooled sample alongside experimental samples. Gels were transferred to PVDF membranes (Cat# 162017, Bio-Rad Laboratories) and briefly stained with Ponceau before blocking in 5% milk for 1 hour. Blots were then incubated overnight at 4°C with primary antibodies: pSMAD3 (1:500 in 2% BSA; Cat# 9520; Cell Signaling Technology), SMAD3 (1:500 in 2% BSA; Cat# MA5-14939; Thermo Fisher Scientific), TGF-βR1 (1:1000 in 5% BSA; Cat# AP14647PU-N; OriGene), and SMAD7 (1:500 in 2% BSA; Cat# MAB2029, R&D Systems). Primary antibodies were detected using a secondary antibody conjugated to horseradish peroxidase: anti-mouse IgG (1:1000 in 5% BSA, Cat# 7076, Cell Signaling Technology) or anti-rabbit IgG (1:1000 in 5% BSA Cat# 7074, Cell Signaling Technology) for 1 hour. Immunoreactions were visualized by SuperSignal™ West Pico PLUS Chemiluminescent Substrate (Cat# 34579, Thermo Fisher Scientific) and imaged using ChemiDoc™ (Bio-Rad Laboratories). Membranes were subsequently stripped for 30 or 45 minutes at room temperature using Restore stripping buffer (21059, Thermo Fisher Scientific), washed, re‑blocked, and probed with the next primary antibody. Each membrane was stripped and reused up to two times. Western blot densitometry quantification was performed using Fiji software
