# Supplementary material for: Aberrant Myelopoiesis and Haematopoietic Stem Cell Niche Remodelling Precedes Cancer Cachexia Onset in a Sex‐Dependent Manner
Source: J Cachexia Sarcopenia Muscle. 2026 Oct 16;17(5):e70403. doi: 10.1002/jcsm.70403 (PMC13673406; doi:10.1002/jcsm.70403)
Supplement: Supplementary file 2 [file JCSM-17-e70403-s005.docx]

**Table S1** Bone Marrow Flow Cytometry Primary Antibody Cocktails

| Antibody Cocktail Description | Antibodies in Cocktail | Dilution of Respective Antibody (5% FBS) | Laser (Conjugate) | Catalogue Number |
| --- | --- | --- | --- | --- |
| HSC | CCR2  Sca1 (Ly-6A/E)  CD150  c-Kit (CD117)  Lineage Panel (5 total)  CD48 | 1:200  1:200  1:200  1:200  1:200(x5)  1:200 | BL1 (FITC)  BL2 (PE)  BL3 (PerCP-eF710)  BL4 (PE-Cy7)  VL1 (BV421)  VL2 (BV510) | 150608  553336  46-1502-82  558163  559971  563536 |
| CMP/GMP /MEP | CCR2  Sca1 (Ly-6A/E)  CD34  c-Kit (CD117)  Lineage Panel (5 total)  CD16/32 | 1:200  1:200  1:200  1:200  1:200(x5)  1:200 | BL1 (FITC)  BL2 (PE)  BL3 (PE-Cy5)  BL4 (PE-Cy7)  VL1 (BV421)  VL4 (BV711) | 150608  553336  119312  558163  559971  101337 |
| CLP | CCR2  Sca1 (Ly-6A/E)  CD135  c-Kit (CD117)  Lineage Panel (5 total)  CD127 | 1:200  1:200  1:200  1:200  1:200(x5)  1:200 | BL1 (FITC)  BL2 (PE)  BL3 (PerCP-eF710)  BL4 (PE-Cy7)  VL1 (BV421)  VL2 (BV510) | 150608  553336  46-1351-82  558163  559971  135033 |
| MSC | CCR2  CD140a  CD45  Ter119  CD31 | 1:200  1:200  1:200  1:200  1:200 | BL1 (FITC)  BL2 (PE)  BL4 (PE-Cy7)  VL1 (BV421)  VL2 (BV510) | 150608  562776  552848  559971  563089 |
| EPCs/ Adipo/Osteo Prog | CCR2  CD51  CD45  Ter119  CD31  Sca1 (Ly-6A/E) | 1:200  1:200  1:200  1:200  1:200  1:200 | BL1 (FITC)  BL2 (PE)  BL4 (PE-Cy7)  VL1 (BV421)  VL2 (BV510)  VL4 (BV711) | 150608  12-0612-82  552848  559971  563089  407-5981-82 |

**Table S2** Tumour Flow Cytometry Primary Antibody Cocktails

| Antibody Cocktail Description | Antibodies in Cocktail | Dilution of Respective Antibody (5% FBS) | Laser (Conjugate) | Catalogue Number |
| --- | --- | --- | --- | --- |
| HSC | CCR2  Sca1 (Ly-6A/E)  CD150  c-Kit (CD117)  Lineage Panel (5 total)  CD48 | 1:200  1:200  1:200  1:200  1:200(x5)  1:200 | BL1 (FITC)  BL2 (PE)  BL3 (PerCP-eF710)  BL4 (PE-Cy7)  VL1 (BV421)  VL2 (BV510) | 150608  553336  46-1502-82  558163  559971  563536 |
| CMP/GMP /MEP | CCR2  Sca1 (Ly-6A/E)  CD34  c-Kit (CD117)  Lineage Panel (5 total)  CD16/32 | 1:200  1:200  1:200  1:200  1:200(x5)  1:200 | BL1 (FITC)  BL2 (PE)  BL3 (PE-Cy5)  BL4 (PE-Cy7)  VL1 (BV421)  VL4 (BV711) | 150608  553336  119312  558163  559971  101337 |
| CLP | CCR2  Sca1 (Ly-6A/E)  CD135  c-Kit (CD117)  Lineage Panel (5 total)  CD127 | 1:200  1:200  1:200  1:200  1:200(x5)  1:200 | BL1 (FITC)  BL2 (PE)  BL3 (PerCP-eF710)  BL4 (PE-Cy7)  VL1 (BV421)  VL2 (BV510) | 150608  553336  46-1351-82  558163  559971  135033 |
| Macrophages (anti- and pro-inflammatory/ Neutrophils/ Monocytes (Classical/ Non-Classical) | CCR2  CD206  CX3CR1  CD45  Ly-6c  Ly-6g  F4/80 | Genetic  1:200  1:200  1:200  1:200  1:200  1:200 | BL1 (eGFP)  BL2 (PE)  BL3 (PerCP-Cy5.5)  BL4 (PE-Cy7)  VL1 (SB436)  VL2 (BV480)  VL4 (SB702) | 12-2061-82  149010  552848  62-5832-82  414-9668-82  67-4801-82 |

**Table S3** Muscle Flow Cytometry Primary Antibody Cocktails

| Antibody Cocktail Description | Antibodies in Cocktail | Dilution of Respective Antibody (5% FBS) | Laser (Conjugate) | Catalogue Number |
| --- | --- | --- | --- | --- |
| CMP/GMP /MEP | CCR2  Sca1 (Ly-6A/E)  CD34  c-Kit (CD117)  Lineage Panel (5 total)  CD16/32 | Genetic  1:200  1:200  1:200  1:200(x5)  1:200 | BL1 (eGFP)  BL2 (PE)  BL3 (PE-Cy5)  BL4 (PE-Cy7)  VL1 (BV421)  VL4 (BV711) | 553336  119312  558163  559971  101337 |
| Macrophages (anti- and pro-inflammatory/ Neutrophils/ Monocytes (Classical/ Non-Classical) | CCR2  CD206  CX3CR1  CD45  Ly-6c  Ly-6g  F4/80 | Genetic  1:200  1:200  1:200  1:200  1:200  1:200 | BL1 (eGFP)  BL2 (PE)  BL3 (PerCP-Cy5.5)  BL4 (PE-Cy7)  VL1 (SB436)  VL2 (BV480)  VL4 (SB702) | 12-2061-82  149010  552848  62-5832-82  414-9668-82  67-4801-82 |

**Table S4** Bone Marrow-Derived Cell Flow Cytometry Gating Strategies

| Cell Population | Gating Strategy |
| --- | --- |
| Hematopoietic Stem Cells (HSCs) | Lineage^-^, Sca-1^+^, c-Kit^+^ (LSK) |
| Long-Term HSCs | LSK, CD48^-^, CD150^+^ |
| Short-Term HSCs | LSK, CD48^-^, CD150^-^ |
| Multipotent Progenitor Cells | LSK, CD48^+^, CD150^-^ |
| Common Myeloid Progenitor Cells | Lineage^-^, Sca-1^-^, c-Kit^+^, CD16/32^int^, CD34^+^ |
| Common Lymphoid Progenitor Cells | LSK, CD135^+^, CD127^+^ |
| Granulocyte-Monocyte Progenitor Cells | Lineage^-^, Sca-1^-^, c-Kit^+^, CD16/32^hi^, CD34^+^ |
| Megakaryocyte-Erythroid Progenitor Cells | Lineage^-^, Sca-1^-^, c-Kit^+^, CD16/32^-^, CD34^-^ |
| Mesenchymal Stromal Cells | Ter119^-^, CD45^-^, CD31^-^, CD140α^+^ |
| Osteoprogenitor Cells | Ter119^-^, Sca-1^+^, CD45^-^, CD51^+^ |
| Osteoblasts | Ter119^-^, Sca-1^-^, CD45^-^, CD51^+^ |
| Adipocyte Progenitor Cells | Ter119^-^, Sca-1^+^, CD45^-^, CD31^-^, CD51^-^ |
| Endothelial Progenitor Cells | Ter119^-^, Sca-1^+^, CD45^-^, CD31^+^ |
| Endothelial Cells | Ter119^-^, CD45^-^, CD31^-^, CD51^-^ |
| Neutrophils | CD45^m/h^, Ly6G^+^ |
| Macrophages | CD45^m/h^, Ly6G^-^, F4/80^+^ |
| M1 Macrophages | CD45^m/h^, Ly6G^-^, F4/80^+^, CD206^-^ |
| M2 Macrophages | CD45^m/h^, Ly6G^-^, F4/80^+^, CD206^+^ |
| Classical Monocytes | CD45^m/h^, Ly6C^Hi^, CCR2^+^, CX3CR1^low^ |
| Non-Classical Monocytes | CD45^m/h^, Ly6C^low^, CCR2^low^, CX3CR1^hi^ |

**Table S5** Percent of Immune Cells Within Skeletal Muscle Across the Experimental Time Course

|  | **Sham** | **2 weeks** | **2.5 weeks** | **3.5 weeks** |
| --- | --- | --- | --- | --- |
|  | Percentage (%) | Percentage (%) | Percentage (%) | Percentage (%) |
| **Neutrophils** | 18.3 | 24.6 | 32.5 | 43.8 |
| **Macrophages** | 22.6 | 23.2 | 19.7 | 21.8 |
| **B-lymphocytes** | 28.3 | 24.4 | 19.3 | 9.6 |
| **Monocytes** | 13.4 | 17.9 | 20.6 | 18.7 |
| **T-lymphocytes** | 15.4 | 8.5 | 4.4 | 2.4 |
| **Megakaryocytes** | 0.9 | 0.6 | 2.4 | 2.7 |
| **Mast Cells** | 1.1 | 0.9 | 1.0 | 0.9 |

**Table S6** Percent of Immune Cells Within Skeletal Muscle That Express Ccr2 Across the Experimental Time Course

|  | **Sham** | **2 weeks** | **2.5 weeks** | **3.5 weeks** |
| --- | --- | --- | --- | --- |
|  | Percentage (%) | Percentage (%) | Percentage (%) | Percentage (%) |
| **Neutrophils** | 0.980392157 | 0.390625 | 0 | 0.616016427 |
| **Macrophages** | 23.01587302 | 29.87551867 | 24.46043165 | 17.3553719 |
| **B-lymphocytes** | 0.0 | 0.0 | 0.0 | 0.9 |
| **Monocytes** | 73.33333333 | 69.89247312 | 54.48275862 | 74.51923077 |
| **T-lymphocytes** | 33.72093023 | 38.63636364 | 35.48387097 | 29.62962963 |
| **Megakaryocytes** | 0.0 | 0.0 | 0.0 | 0.0 |
| **Mast Cells** | 33.33333333 | 77.77777778 | 14.28571429 | 10 |

**Table S7** Percent of Cells Within Skeletal Muscle That Express Ccl2 Across the Experimental Time Course

|  | Sham | 2-weeks | 2.5-weeks | 3.5-weeks |
| --- | --- | --- | --- | --- |
|  | Proportion (%) | Proportion (%) | Proportion (%) | Proportion (%) |
| Endothelial cells | 0.13 | 0.11 | 0.25 | 1.92 |
| Myonuclei | 0.00 | 0.12 | 0.06 | 2.08 |
| FAPs | 1.83 | 1.46 | 1.94 | 6.73 |
| MuSCs | 0.37 | 0.69 | 0.00 | 4.42 |
| Neutrophils | 0.00 | 0.78 | 0.00 | 5.54 |
| Macrophages | 16.67 | 26.14 | 20.86 | 49.59 |
| Monocytes | 5.33 | 9.68 | 6.90 | 26.92 |
| Pericytes | 0.34 | 1.15 | 1.43 | 5.50 |
| B-lymphocytes | 0.00 | 0.79 | 0.00 | 0.00 |
| vSCMs | 0.00 | 0.00 | 0.68 | 2.63 |
| Tenocytes | 0.88 | 0.72 | 1.10 | 3.65 |
| Gilal Cells | 0.96 | 0.00 | 0.00 | 1.64 |
| Schwan Cells | 0.00 | 0.00 | 0.00 | 6.19 |
| T-lymphocytes | 0.00 | 26.92 | 0.00 | 0.00 |
| Megakaryocytes | 0.00 | 0.00 | 0.00 | 0.00 |
| Mast Cells | 0.00 | 11.11 | 28.57 | 90.00 |
